# Case Report: Long-lasting adrenal insufficiency following successful osilodrostat therapy with subsequent recurrence of Cushing’s syndrome

**DOI:** 10.3389/fendo.2026.1962865

**Published:** 2026-10-14

**Authors:** Wiktoria Suchy, Ewelina Rzepka, Mari Minasyan, Aleksandra Gamrat-Żmuda, Alicja Hubalewska-Dydejczyk, Aleksandra Gilis-Januszewska

**Affiliations:** Chair and Department of Endocrinology, Jagiellonian University Medical College, Krakow, Poland

**Keywords:** adrenal Insufficiency, bilateral inferior petrosal sinus sampling, case report, Cushing’s syndrome, osilodrostat

## Abstract

Osilodrostat is an effective steroidogenesis inhibitor used in the treatment of Cushing’s syndrome (CS). One of the clinically significant adverse events associated with this therapy is adrenal insufficiency (AI). Here, we report a patient with CS who developed delayed-onset AI during osilodrostat therapy, that persisted for four years despite drug discontinuation and was followed by recurrence of hypercortisolism. A 52-year-old female was admitted with suspicion of CS. Diagnostic evaluation suggested pituitary CS. Ketoconazole was initiated as a preoperative treatment, however, transsphenoidal surgery (TSS) proved non-radical. The patient declined reoperation, and was therefore treated with metyrapone within a clinical trial (NCT02297945), followed by transition to osilodrostat after the trial’s completion. After two years of successful treatment with a stable dose of osilodrostat the patient developed AI. The drug was discontinued, however AI persisted for the next 4 years. The symptoms suggestive of CS recurred, which led to another TSS, that resulted in remission. After 10 months of remission, hypercortisolism recurred again. This case highlights the need for careful long-term monitoring in patients receiving adrenostatic therapy. It is particularly notable for delayed onset of AI, despite long-term treatment with a stable, relatively low dose of osilodrostat. The prolonged persistence of AI and the subsequent late recurrence of hypercortisolism further distinguish this case from previously reported cases. Overall, this case underscores the need for continued clinical vigilance during long-term osilodrostat treatment.

## 1 Introduction

Osilodrostat is a potent and effective adrenal steroidogenesis inhibitor used in the treatment of Cushing’s syndrome (CS). It acts by blocking 11β-hydroxylase, an enzyme responsible for the last step of cortisol synthesis (1). Among currently available medical therapies for CS, osilodrostat demonstrates one of the highest rates of biochemical control (2). One of the rare, but clinically significant adverse events associated with the use of this drug is long-lasting adrenal insufficiency (AI) (3–6). AI occurs most commonly during treatment initiation, however, delayed-onset cases have also been reported (7). Moreover, this can last even after the therapy has been terminated. Here, we report a case of delayed-onset AI during osilodrostat therapy, which persisted after drug discontinuation and was followed by recurrence of CS.

## 2 Case presentation

### 2.1 Diagnosis

A 52-year-old female was admitted to the Endocrinology Department with suspicion of hypercortisolism. She was diagnosed with hypertension 7 months earlier and presented with face plethora, moon face, central obesity, easy bruising, and muscle wasting that started 6 months earlier. Moreover, she reported mood swings and sleeplessness. During the initial diagnostic work-up, an oral glucose tolerance test was performed, leading to the diagnosis of diabetes and insulin resistance. Biochemical assessment confirmed adrenocorticotropic hormone (ACTH)-dependent CS and the 8-mg dexamethasone suppression test supported pituitary origin of ACTH overproduction (**Table 1A**). The patient initially received ketoconazole at a dose of 600 mg daily. Due to hepatotoxicity, with liver enzymes elevated up to four times the upper limit of normal, the dose was reduced to 400 mg daily. Pituitary MRI revealed a hypointense lesion measuring approximately 2.7 mm in the left side of the anterior pituitary lobe, consistent with a microadenoma. Therefore, the patient underwent transsphenoidal surgery (TSS). Following TSS, cortisol concentrations remained elevated, with persistent hypertension. Histopathological examination revealed the presence of pituitary tissue and fragments of a pituitary adenoma. Immunohistochemical staining showed weak ACTH expression, with Ki-67 <1%. Despite the surgery being non-radical, the patient declined immediate reoperation. Follow-up pituitary MRI performed 3 months after TSS no longer demonstrated the previously described focal lesion. Meanwhile, a corticotropin-releasing hormone (CRH) stimulation test was performed, consistently supporting the diagnosis of Cushing’s disease. Cortisol increased from 27.63 µg/dl to a peak of 35.00 µg/dl at 180 minutes (27% increase), while ACTH increased from 179 pg/ml to a maximum of 332 pg/ml at 15 minutes (85% increase).

**Table 1:** Biochemical evaluation at the time of: A, first admission; B, adrenal insufficiency; C, adrenal function recovery; D, recurrence.

Test	Result	
A. Diagnosis		
Serum cortisol concentration (8AM)	30.2 µg/dl (N: 4.82-19.5)	
Serum cortisol concentration (12AM)	24.4 µg/dl (N: <7.5)	
Serum ACTH concentration (8AM)	91.0 pg/ml (N: 6.0-56.0)	
Serum cortisol concentration after 1 mg DST	15.8 µg/dl (N: <1.8)	
Serum cortisol concentration after 8 mg DST	26.5 µg/dl → 7.22 µg/dl (73% decrease)	
Urinary free cortisol	205.8 µg/24h (N: 10-100)	
Serum DHEA-S concentration (8AM)	3.83 µmol/l (N: 0.51-5.56)	
B. Adrenal insufficiency		
Serum cortisol concentration (8AM)	0.38 µg/dl (N: 4.82-19.5)	
Serum ACTH concentration (8AM)	66.3 pg/ml (N: 6.0-56.0)	
Serum DHEA-S concentration (8AM)	0.09 µmol/l (N: 0.51-5.56)	
C. Adrenal function recovery		
Serum cortisol concentration in tetracosactide stimulation test (250 µg)	0 min	11.6 µg/dl
	30 min	15.9 µg/dl
	60 min	17.6 µg/dl
	120 min	18.0 µg/dl
Serum ACTH concentration (8AM)	209.5 pg/ml (N: 6.0-56.0)	
Serum DHEA-S concentration (8AM)	0.18 µmol/l (N: 0.51-5.56)	
D. Recurrence		
Serum cortisol concentration (8AM)	15.6 µg/dl (N: 4.82-19.5)	
Serum cortisol concentration (12AM)	15.1 µg/dl (N: <7.5)	
Serum ACTH concentration (8AM)	234.2 pg/ml (N: 6.0-56.0)	
Serum cortisol concentration after 1 mg DST	11.8 µg/dl (N: <1.8)	
Late-night salivary cortisol	0.47 µg/dl (N: <0.274)	
Urinary free cortisol	117.9 µg/24h (N: 10-100)	

ACTH, adrenocorticotropic hormone; DHEA-S, dehydroepiandrosterone sulfate; DST, dexamethasone suppression test.

Due to insufficient biochemical control, the ketoconazole dose was increased to 600 mg daily, however, the hepatotoxicity recurred, which led to eventual drug discontinuation after 6 months of treatment. Two months later, medical therapy with metyrapone was initiated at a dose of 1500 mg daily and subsequently titrated (750–1750 mg daily), according to the study protocol (8). After 36 weeks of therapy, despite optimal biochemical control and good compliance, the drug was discontinued due to the study termination. Subsequently, the patient started receiving osilodrostat at a dose of 2 mg twice daily, according to the study protocol (NCT03606408). This resulted in substantial biochemical and clinical improvement with an increase in muscle strength from approximately 40% to 75–90% of normal function (Lovett scale score from 3 to 4).

Just before inclusion in the study, the patient’s numerous repeated pituitary MRI scans were reviewed by a radiologist. The specialist concluded that across all evaluated scans, a small residual area of tissue, measuring up to approximately 5 mm, persists in the medial aspect of the right cavernous sinus, raising suspicion of a microadenoma in the context of compatible clinical findings.

### 2.2 Adrenal insufficiency

Two years after treatment initiation, while receiving a stable daily dose of 4 mg, the patient reported symptoms such as nausea, abdominal pain, myalgia, and fatigue. Blood pressure values measured at home were 98/67 mmHg, with heart rate over 100 beats per minute.

The patient was informed to discontinue the drug, take 20 mg of hydrocortisone orally and visit the site the next morning. Despite the actions taken by the patient, the symptoms persisted. Dosing errors were excluded with documented treatment adherence of 98% since the previous visit. During the hospitalization, AI was biochemically confirmed with a morning serum cortisol concentration of 0.38 µg/dl (**Table 1B**). After adjusting the dose of hydrocortisone to 25 mg daily, the patient’s condition significantly improved. Following discharge, she only occasionally required hydrocortisone dosage adjustments. During 4 years of follow-up, AI persisted, with a gradual increase in morning serum cortisol concentrations up to 12.2 µg/dl. During this period, the patient underwent two abdominal MRI scans and one abdominal CT scan (primarily for evaluation of hepatic cysts), none of which revealed any adrenal pathology. Repeated pituitary MRI scans performed 4, 22, and 44 months after AI onset consistently showed a heterogenous signal of the anterior lobe, with no unambiguous evidence of disease recurrence.

### 2.3 Adrenal function recovery

Fifty months after the AI incidence, tetracosactide stimulation test was performed (**Table 1C**), and confirmed recovery of adrenal reserve sufficient to discontinue routine hydrocortisone replacement, while maintaining the *ad hoc* supplementation.

### 2.4 Recurrence

Six months later, the patient presented with weight gain, progressive muscle weakness, and worsening blood pressure control, which raised suspicion of disease relapse. Biochemical evaluation confirmed recurrence of CS (**Table 1D**) and desmopressin test again suggested pituitary origin, with cortisol increasing from 14.3 µg/dl to a peak of 17.6 µg/dl at 30 minutes (23% increase), and ACTH increasing from 179 pg/ml to a maximum of 416 pg/ml at 30 minutes (132% increase). Pituitary MRI revealed heterogenous signal of the anterior lobe, with loss of parenchyma in the left-sided portion, consistent with prior surgery. Bilateral inferior petrosal sinus sampling (BIPSS) was performed, confirming Cushing’s disease.

This led to another TSS, which resulted in CS remission. Histopathological examination revealed fragments of pituitary tissue and fibrous tissue, along with several small fragments of a corticotroph pituitary adenoma. Immunohistochemical studies showed ACTH expression, with Ki-67 <3%. After 10 months of remission, the patient experienced another recurrence, and is currently awaiting enrollment in a treatment program. The clinical course and treatment modalities are presented in **Figure 1** and in the **Supplementary Figure S1**.

**Figure 1:**
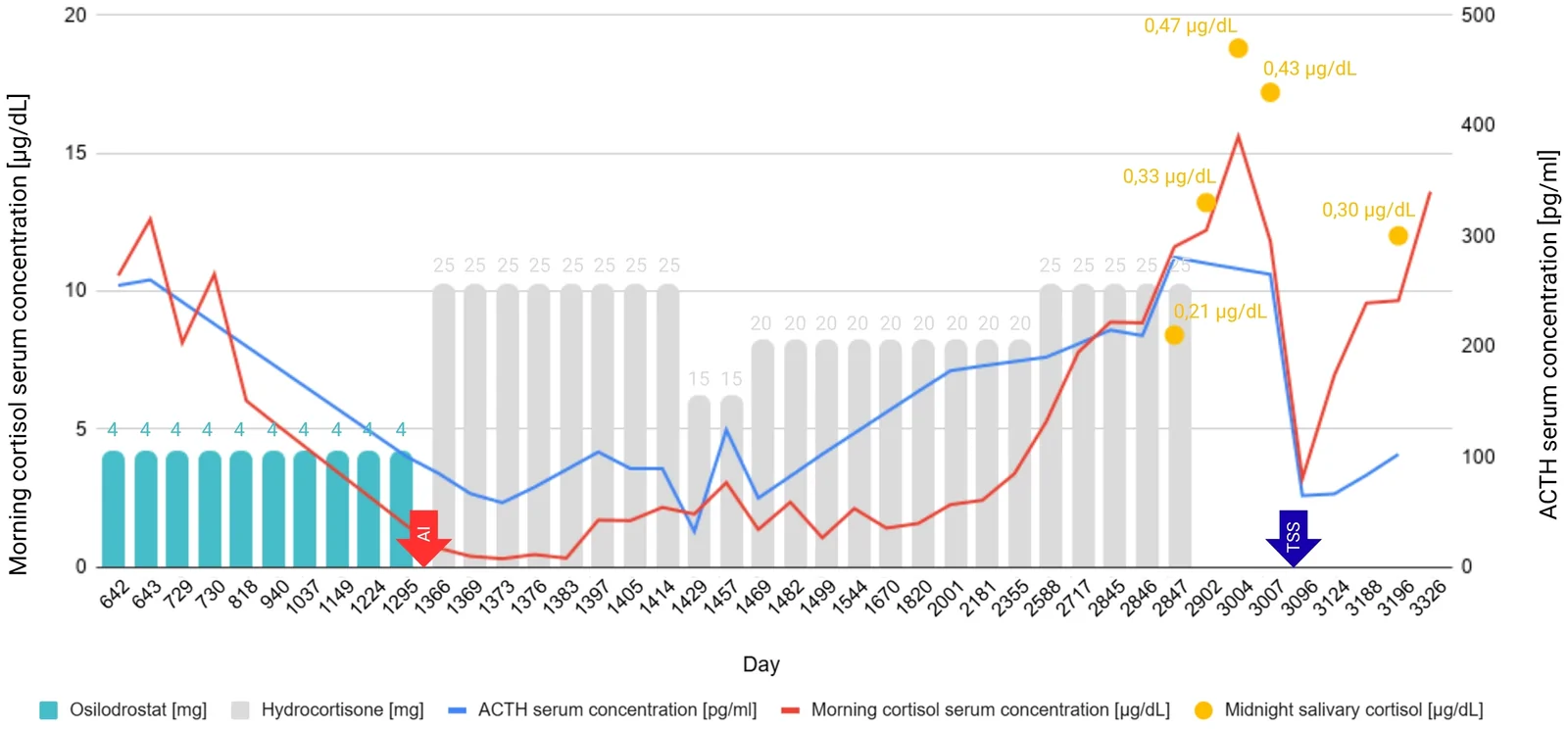
Overview of clinical course and treatment following osilodrostat initiation. AI, adrenal insufficiency; TSS, transsphenoidal surgery.

## 3 Discussion

Hypocortisolism-related adverse effects are frequent during osilodrostat therapy. However, the majority of them are mild or moderate, very rarely leading to permanent drug discontinuation (3, 4). AI is at the end of this spectrum, remaining one of the most serious and potentially life-threatening adverse effects of the drug. Although usually associated with the period shortly after treatment initiation, it can occur at any time (7). In our patient, despite a stable dose, AI occurred 2 years after osilodrostat introduction. Importantly, AI developed despite long-term treatment with a stable low dose of osilodrostat and without recent dose escalation, which distinguishes our case from the majority of previously reported cases.

Prolonged AI, especially followed by recurrence of CS, is extremely rare. Typically, the symptoms subside after drug discontinuation. To date, eleven cases of AI persisting despite osilodrostat therapy termination have been reported (maximum daily doses: 2-20 mg) (6, 9–15), with only three cases of subsequent CS recurrence (6, 9). Summary of the cases is presented in **Table 2**. In our patient, delayed AI occurred after two years of successful treatment, which is also extraordinary, since the time from drug introduction to first AI episode in reported cases ranged from 1 week to 18 months (6, 9–15). Notably, CS recurrence in our case occurred 56 months after AI incidence, significantly later than in other reported cases (1.5–14 months) (6, 9).

**Table 2:** Summary of reported cases of osilodrostat-associated prolonged adrenal insufficiency.

Article	Age (years)	Sex	CS etiology	Maximum osilodrostat dose (mg/day)	Time to first AI (months)	Total duration of osilodrostat treatment (months)	Duration of AI (months)	Time from AI to CS relapse (months)
Działach et al. (6)	41	M	PIT-CS	10	5	63	5	5
Poirier et al. (9)	51	F	PIT-CS	2	6	6	1.5	1.5
Poirier et al. (9)	31	F	PIT-CS	4	15	15	14	14
Poirier et al. (9)	41	M	ACTH-dependent	10	13	13	10*	–
Tejani et al. (10)	41	F	PIT-CS	6	11	11	23	–
Ferriere et al. (11)	59	M	ECT-CS	20	0.25	6	12	–
Ferriere et al. (11)	51	F	PIT-CS	10	2.5	15	15*	–
Kaniuka-Jakubowska et al. (12)	74	F	ECT-CS	4	1	15	11*	–
Veloski et al. (13)	38	F	ADR-CS	20	4	4	36*	–
Sasson et al. (14)	70	F	PIT-CS	4	0.33	0.33	3	–
Sofer et al. (15)	70	M	PIT-CS	20	18	18	30*	–
Our patient	55	F	PIT-CS	4	24	24	50	56

*AI at the time of article publication.

AI, adrenal insufficiency; ACTH, adrenocorticotropic hormone; ADR-CS, adrenal Cushing’s syndrome; CS, Cushing’s syndrome; ECT-CS, ectopic Cushing’s syndrome; F, female; M, male; PIT-CS, pituitary Cushing’s syndrome.

The mechanisms underlying persistent AI remain unclear and seem counterintuitive considering the relatively short half-life of osilodrostat (4 hours) (1). Several hypotheses have been proposed, including the effect of cumulative dose of the drug, as well as possible impact on enzymatic steps other than 11β-hydroxylase (6, 12). Notably, we observed a marked reduction in dehydroepiandrosterone sulfate (DHEA-S) concentration following the AI incidence. This finding, together with previous *in vitro* studies suggesting that osilodrostat may affect 17α-hydroxylase/17,20-lyase (17α-OH) activity, further supports the possibility that osilodrostat may influence additional steps of steroidogenesis (16). Finally, some researchers point to adrenal gland shrinkage in some patients treated with osilodrostat, which could indicate a possible adrenolytic effect of the drug (2, 17). However, repeated imaging in our patient did not reveal any adrenal abnormalities.

Taken together, our case underscores the importance of long-term follow-up in patients receiving adrenostatic therapy, which applies not only to those shortly after drug introduction or recent dose increase, but also to patients remaining on a stable dose for a long time. The exceptionally delayed onset of AI after two years of stable osilodrostat treatment, its prolonged persistence, and the very late recurrence of CS 56 months after the initial episode distinguish this case from previously reported cases and make it particularly relevant to the long-term management of patients receiving osilodrostat. Moreover, it highlights the crucial role of patient education and awareness of symptoms suggestive of AI along with knowledge about appropriate hydrocortisone replacement in the context of suspected hypocortisolism. We would also like to emphasize that careful longitudinal monitoring of patients with drug-related AI is also essential to detect adrenal function recovery or recurrence of CS.

## Data Availability

The original contributions presented in the study are included in the article/**Supplementary Material**. Further inquiries can be directed to the corresponding author.
